# Supplementary material for: UXT chaperone prevents proteotoxicity by acting as an autophagy adaptor for p62-dependent aggrephagy
Source: Nat Commun. 2021 Mar 29;12:1955. doi: 10.1038/s41467-021-22252-7 (PMC8007730; doi:10.1038/s41467-021-22252-7)
Supplement: Supplementary file 1 — Supplementary Information [file 41467_2021_22252_MOESM1_ESM.pdf]

Prey ID	Description	Reporter expression		
		lacZ	*URA3*	*ADE2*
**AD-Hybrid - 1**	The activation domain (AD) is fused in frame to the 14th aa of alcohol dehydrogenase 1B (class I), beta polypeptide (**ADH1B**), transcript variant 2 (NM_001286650).	-	+	+
**AD-Hybrid - 2**	The activation domain (AD) is fused in frame to the 1170th aa of SET domain, bifurcated 1 (**SETDB1**), transcript variant 1 (NM_001145415.1).	-	+	+
**AD-Hybrid - 3**	The activation domain (AD) is fused in frame to the 99th aa of succinate dehydrogenase complex iron sulfur subunit B (**SDHB**) (NM_003000).	-	+	+
**AD-Hybrid - 4**	The activation domain (AD) is fused in frame to the 141st aa of succinate dehydrogenase complex iron sulfur subunit B (**SDHB**) (NM_003000).	+	+	+
**AD-Hybrid - 5**	The activation domain (AD) is fused to 5' UTR (untranslated region) of ubiquitously expressed prefoldin like chaperone (**UXT**), transcript variant 2 mRNA at -15nt (NM_004182).	+	+	+
**AD hybrid - 6,7**	The activation domain (AD) is fused to 5' UTR of ubiquitously expressed prefoldin like chaperone (**UXT**), transcript variant 2 mRNA at -3 nt (NM_004182).	+/-	+	+
**AD hybrid - 8**	The activation domain (AD) is fused to 5' UTR of ubiquitously expressed prefoldin like chaperone (**UXT**), transcript variant 2 mRNA at -12 nt (NM_004182).	+	+	+
**AD hybrid - 9-13**	The activation domain (AD) is fused to ubiquitously expressed prefoldin like chaperone (**UXT**), transcript variant 2 (NM_004182).	+/-	+	+
**AD hybrid - 14,15**	The activation domain (AD) is fused to the sequence encoding vimentin which is followed by tubulin beta class I encoding sequence.	-	+	+
**AD hybrid - 16**	The activation domain (AD) is fused in frame to the 89th aa of nephronophthisis 3 (adolescent) (**NPHP3**) (NM_153240).	-	+	+
**AD-Hybrid -17,18**	The activation domain (AD) is fused to nephronophthisis 3 (adolescent) (NPHP3) (NM_153240).	-	+	+
**AD-Hybrid - 19**	The activation domain (AD) is fused in frame to the 3rd aa of enoyl-CoA hydratase, short chain, 1, mitochondrial (**ECHS1**) (NM_004092).	-	+	+
**AD-Hybrid - 20**	The activation domain (AD) is fused in frame to the 34th aa of regulatory factor X associated ankyrin containing protein (**RFXANK**), transcript variant 1 (NM_003721).	-	+	+
**AD-Hybrid - 21**	The activation domain (AD) is fused in frame to the 16th aa of zinc finger protein 426 (**ZNF426**), transcript variant 1 (NM_024106).	-	+	+
**AD-Hybrid - 22,23**	The activation domain (AD) is fused in frame to the 2nd aa of glycine cleavage system protein H (aminomethyl carrier) (**GCSH**), transcript variant 1 (NM_004483).	-	+	+
**AD hybrid - 24**	The activation domain (AD) is fused in frame to the 73rd aa of proteasome subunit beta 5 (**PSMB5**), transcript variant 1 (NM_002797).	-	+	+
**AD hybrid - 25**	The activation domain (AD) is fused in frame to the 265th aa of carboxypeptidase B1 (**CPB1**) (NM_001871).	-	+	+
**AD hybrid - 26**	The activation domain (AD) is fused out of frame to the 450th aa of EWS RNA-binding protein 1 (**EWSR1**), transcript variant 4 (NM_001163286).	-	+	+
**AD hybrid - 27**	The activation domain (AD) is fused to cDNA FLJ39437 fis, clone PROST2005067 (AK096756).	-	+	+

Supplementary Table 1. List of clones identified from yeast two hybrid screening with p62(ZZ-LB) as a bait.

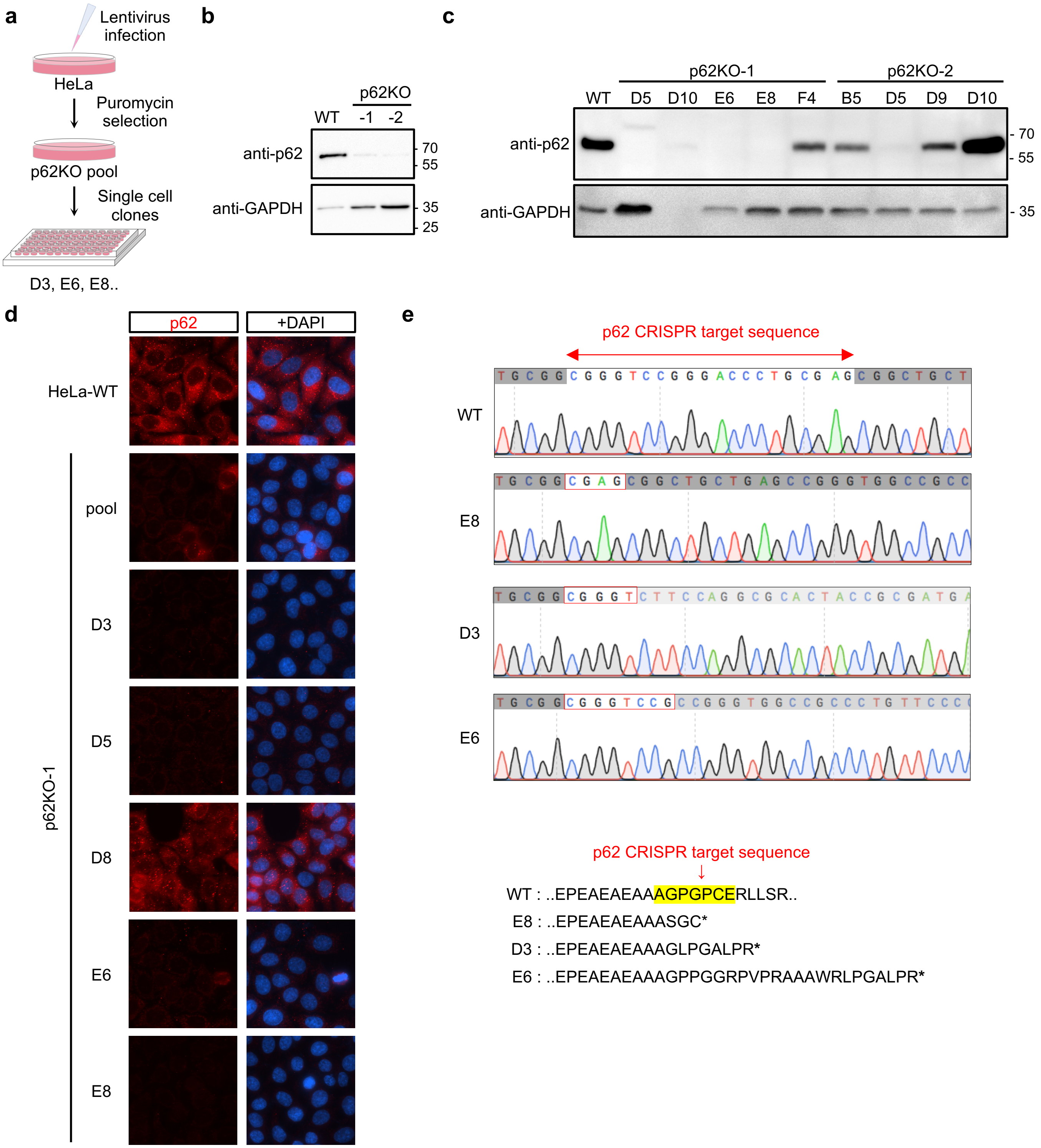

Supplementary Figure 1. Knock out of p62 in HeLa cells using a CRISPR/Cas9 system.
a Two different 20 bp-target single-guide RNA sequences were obtained using CRISPR Design online software (http://crispr.mit.edu/). Target guide RNAs were cloned into a lentiCRISPRv2 backbone that was used for generating lentiviruses. The resulting two types of viral particles were used to transduce HeLa cells. After puromycin selection, two p62KO pools, named p62KO-1 and -2, were generated. From these pooled cells, single cell clones were generated, and their genomic sequences were verified. **b** The expression of p62 in p62KO-1 and -2 cells was verified by western blotting with anti-p62. **c, d** Single cell clones D3, D5, D8, D10, E6, E8, F4 from p62KO-1 cells, and B5, D5, D9, and D10 from p62KO-2 cells were generated. p62 expression in the clones was verified by (**c**) western blotting (**d**) and/or cell staining with anti-p62 followed by anti-mouse IgG-TRITC. **e** p62 gene sequences in the clones were analyzed and premature stop codons were confirmed. The resulting E8 clone was used as HeLa/p62KO cells in this study.

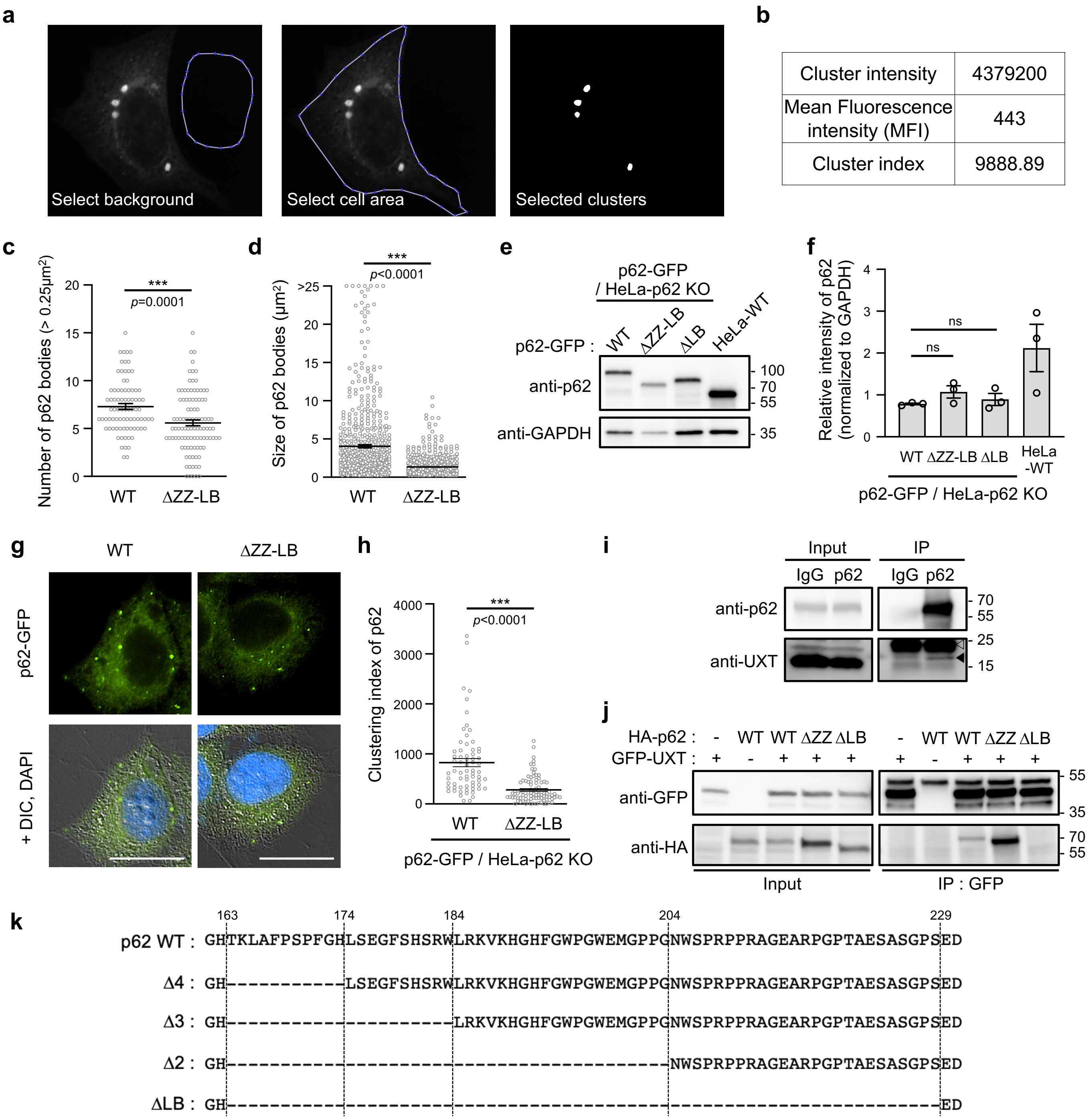

Supplementary Figure 2. The LB domain-dependent interaction of p62 with UXT.
a An example illustrating the measurement of the degree of clustering by using supplementary Code 1. The original images with manually selected background (left), cell region (middle) and the binary image showing the automatically selected clusters/aggregates (right) are shown. **b** The calculated sum of pixel intensities of the selected clusters/aggregates (cluster intensity), the mean fluorescence intensity of the cell region (MFI) and the cluster/aggregation index of the cell in (**a**) are shown. **c, d** In the fluorescence images used for Fig. 1c, the number of p62 bodies per cell with area > 0.25 μm^2 and the size of each p62 bodies were measured (n = 82 (WT) and 101 (ΔZZ-LB) cells, mean ± SEM). ***, p < 0.001 (two-sided Mann-Whitney test). **e** HeLa/p62KO cells stably expressing p62 wild type-, ΔZZ-LB or ΔLB mutant-GFP were generated using lentiviral transduction followed by sorting the GFP-positive cells with flow cytometry, and p62 expression levels were compared by western blotting. **f** Quantitative analysis of (**e**) (n = 3, mean ± SEM). ns, not significant (two-sided unpaired t-test). **g, h** p62 body formation of HeLa/p62KO cells stably expressing p62- or p62(ΔZZ-LB)-GFP were compared. Representative images are shown in (**g**) and analyzed clustering indexes are shown as scatter plots in (**h**). n = 67 (WT) and 89 (ΔZZ-LB) cells, mean ± SEM. ***, p < 0.001 (two-sided Mann-Whitney test). **i** HeLa cells fixed with 0.5% formaldehyde were lysed with RIPA buffer (25 mM Tris, pH 7.6, 150 mM NaCl, 0.1 % SDS, 1 % sodium deoxycholate, 1 % NP-40), and then clarified by centrifugation. Resulting lysates were proceeded to immunoprecipitation using normal rabbit IgG or anti-p62 antibody. Cross-linked proteins in the p62 precipitates were released by heating and analyzed by western blots. Empty and filled black arrowheads indicate IgG light chain and UXT, respectively. **j** Interactions between GFP-UXT and HA-p62 wild type or mutants were analyzed. **k** Amino acid sequences of p62 LB domain and its deletion mutants used in Fig. 1j.

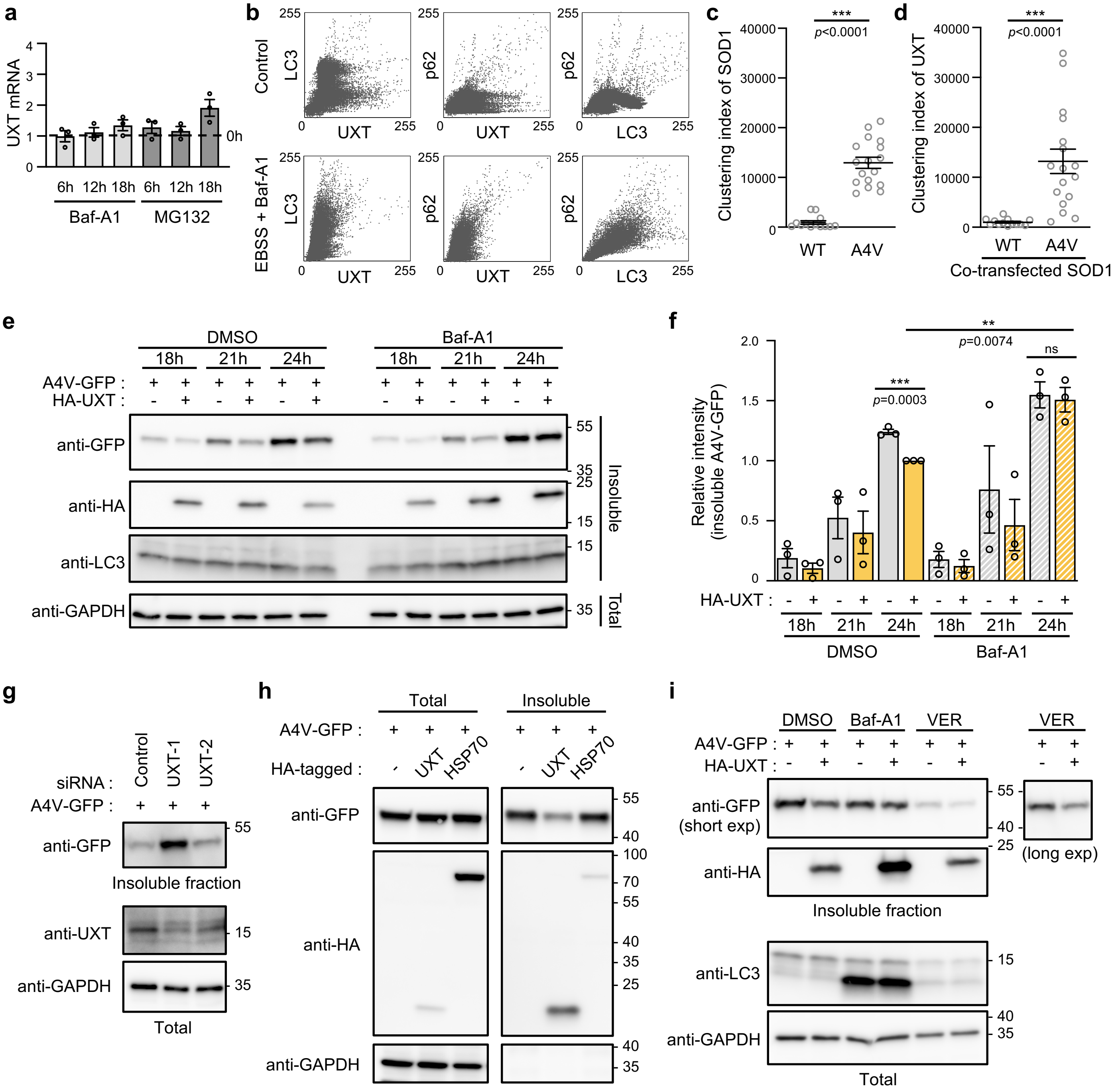

Supplementary Figure 3. Autophagic clearance of SOD1(A4V) aggregates by UXT.

a HeLa cells were treated with 50 nM Baf-A1 or 2.5 μM MG132 for 6, 12, or 18 h and UXT mRNA levels were analyzed by quantitative reverse-transcription polymerase chain reaction. UXT mRNA levels were normalized to that of actin and are shown as bar graphs. Error bars indicate standard error (n = 3). **b** Colocalization of UXT, LC3, and p62 in the region indicated with boxes in Fig. 3d was visualized by plotting their pixel intensities in the scatter plot. **c, d** HEK293T cells were transiently transfected with SOD1-GFP or SOD1(A4V)-GFP with mCherry-UXT or mCherry-vector control. **c** Clustering indexes of SOD1 WT-GFP or SOD1(A4V)-GFP were compared (n = 13 and 17 cells for SOD1 WT and SOD1(A4V), mean ± SEM). ***, p<0.001 (two-sided Mann-Whitney test). **d** Clustering indexes of UXT in wild type or A4V mutant SOD1-transfected cells are shown. Mean ± SEM is indicated. ***, p<0.001 (two-sided Mann-Whitney test). **e** HEK293T cells transfected with SOD1(A4V)-GFP and HA-UXT were treated with 50nM Baf-A1 17 h after transfection. These cells were harvested at the indicated time, and insoluble fractions as well as whole lysates were subjected to a western blot analysis. **f** Band intensities of insoluble SOD1(A4V)-GFP in (**e**) were normalized against that of HA-UXT-transfected cells without Baf-A1 treatment at 24h and are shown as bar graphs (n = 3, mean ± SEM). **, p < 0.01; ***, p <0.001; ns, not significant (two-sided unpaired t-test). **g** HeLa cells were transfected with siRNAs against UXT, UXT-1 and -2, or control siRNA. 48h after transfection, SOD1(A4V)-GFP construct was transfected further to these cells and incubated for 24h. The level of SOD1(A4V)-GFP in the detergent-insoluble fraction was compared. **h** HEK293T cells were transfected with SOD1(A4V)-GFP and HA-UXT or HA-HSP70, and SOD1(A4V)-GFP level in the detergent-insoluble fraction was analyzed by western blot. **i** HEK293T cells were transfected with SOD1(A4V)-GFP and HA-UXT as indicated. After 24h of transfection, 50nM bafilomycin A1 or 30μM VER155008 (VER) was treated for 16h and insoluble SOD1(A4V)-GFP level was analyzed by western blot.

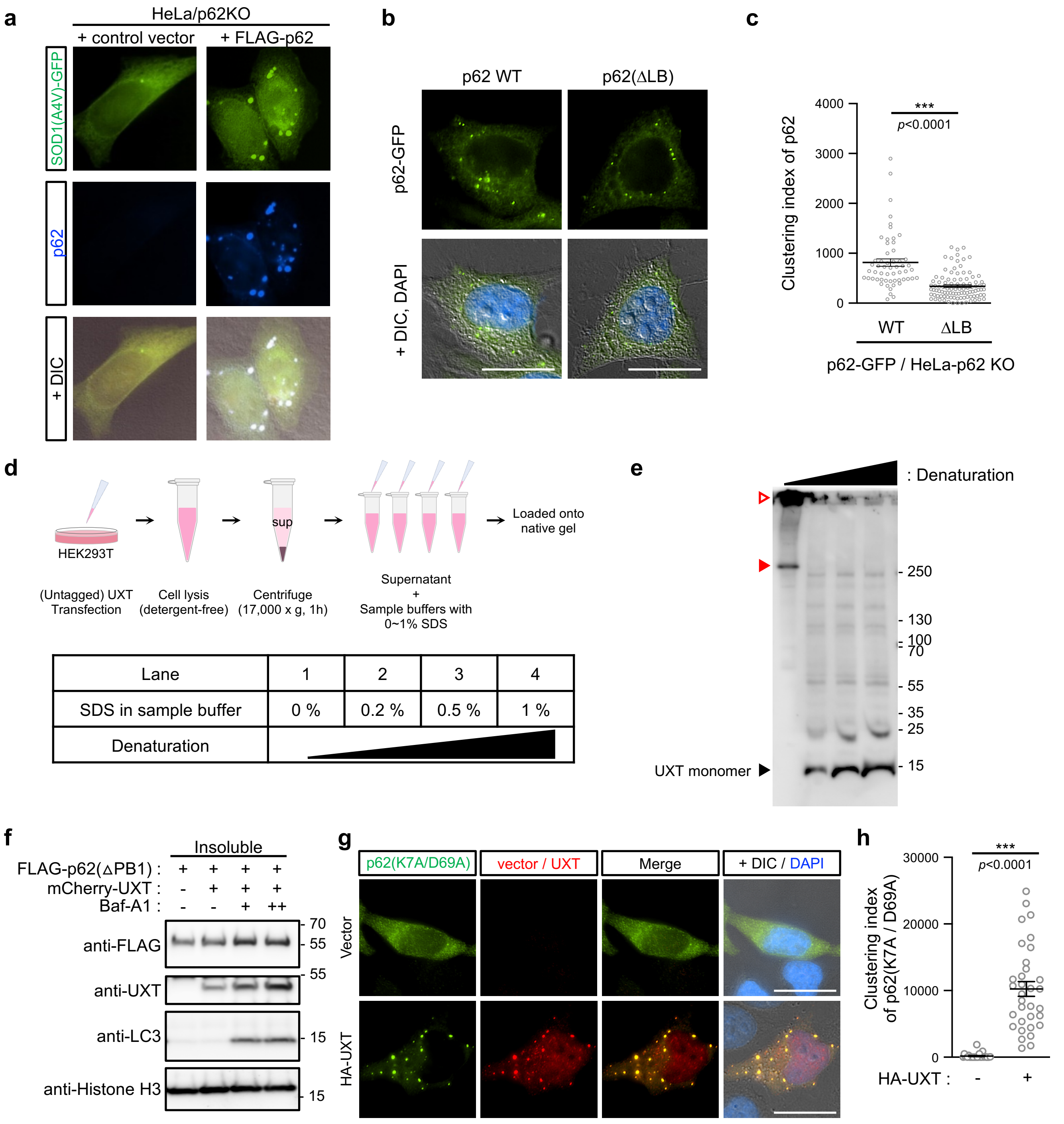

Supplementary Figure 4. UXT-dependent oligomerization of p62.

a Suppression of SOD1 (A4V) aggregates formation in HeLa/p62KO cells. HeLa/p62KO cells transfected with SOD1(A4V)-GFP and FLAG-p62 or vector-control were treated with 5 µM MG132 for 6 h. The cells were stained with anti-p62, anti-mouse IgG-Alexa-350. Representative images are shown. Note that without p62 expression, SOD1(A4V) is diffusely localized throughout the cytoplasm and does not form punctate structures. **b, c** p62 body formation of HeLa/p62KO cells stably expressing p62- or p62(ΔLB)-GFP were compared as in supplementary Fig. 2g-h. (n = 56 and 97 cells for wild type and ΔLB mutant cells, mean ± SEM). ***, $p < 0.001$ (two-sided Mann-Whitney test). **d** HEK293T cells were transfected with untagged-UXT. Detergent-free cell lysates were prepared and mixed with SDS-containing sample buffer as indicated. **e** Samples prepared as in (**d**) were subjected to a native PAGE analysis followed by western blot using anti-UXT antibody. UXT signals were observed as large aggregates stuck on the well (empty red triangle) and at near 250 kDa marker (filled red triangle). **f** HeLa/p62KO cells were transiently transfected with FLAG-p62(ΔPB1) and mCherry-UXT, and at 18h after transfection, 50nM Baf-A1 was treated for 6h and detergent insoluble fractions were subjected to a western blot analysis. **g** HeLa/p62KO cells were transiently transfected with oligomerization-defective p62(K7A/D69A)-GFP and HA-UXT. The cells were stained with anti-HA antibody followed by anti-rabbit IgG-TRITC. The representative images are shown. Note that overexpression of HA-UXT induces formation of p62 body-like structure of p62(K7A/D69A). **h** Clustering indexes of cells in (**g**) were shown as scatter plots (n = 27 (p62 K7A/D69A without UXT) and 33 (p62 K7A/D69A with UXT) cells, mean ± SEM). ***, $p < 0.001$ (two-sided Mann-Whitney test).

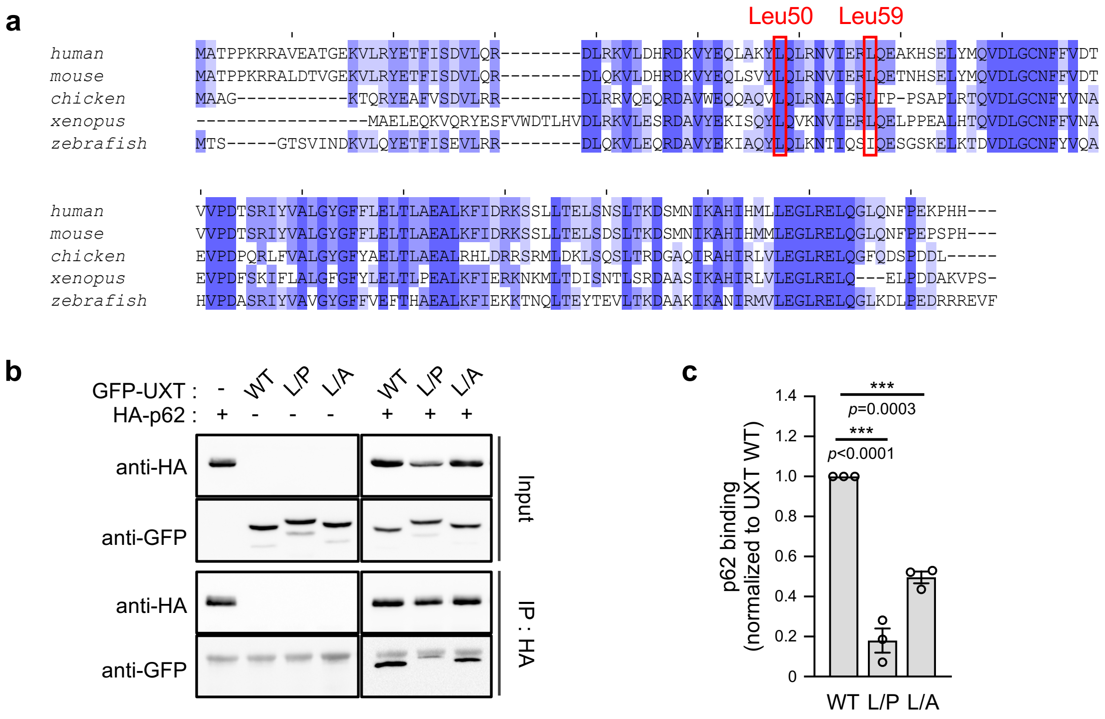

Supplementary Figure 5. p62 binding-defective UXT mutants.

a Sequence alignment of UXT in vertebrates. Amino acid sequences of Homo sapiens (human), Mus musculus (mouse), Gallus gallus (chicken), Xenopus tropicalis (xenopus), and Danio rerio (zebrafish) were aligned using BLASTp (https://blast.ncbi.nlm.nih.gov/). UXT is conserved in vertebrates. Human UXT shows high sequence identity (143/157, 91%) and similarity (149/157, 94%) with no gaps (0/157, 0%) to mouse; and similarly to chicken (82/138 (59%) identity; 105/138 (76%) similarity; 1/138 (0%) gap); xenopus (85/141 (60%) identity; 116/141 (82%) similarity; 0/141 (0%) gap); and zebrafish (89/139 (64%) identity; 115/139 (82%) similarity; 0/139 (0%) gap). Amino acids that are required for binding to p62 (L50 and L59) are conserved in all vertebrates. According to the number of identical residues among species, background of each residue is colored by dark blue (5), blue (4), or light blue (3). **b** Interactions between HA-p62 and GFP-UXT (wild type or L50P/L59P(L/P) and L50A/L59A (L/A) mutants) were analyzed by detection of GFP-UXT in precipitates of HA-p62. **c** Quantitative analysis of (**b**). Band intensities of the UXT mutants in HA-p62 precipitates were normalized against that of the UXT wild type in HA-p62 precipitates. (n = 3, mean ± SEM). ***, $p < 0.001$ (one-way ANOVA using Bonferroni's multiple comparison test).

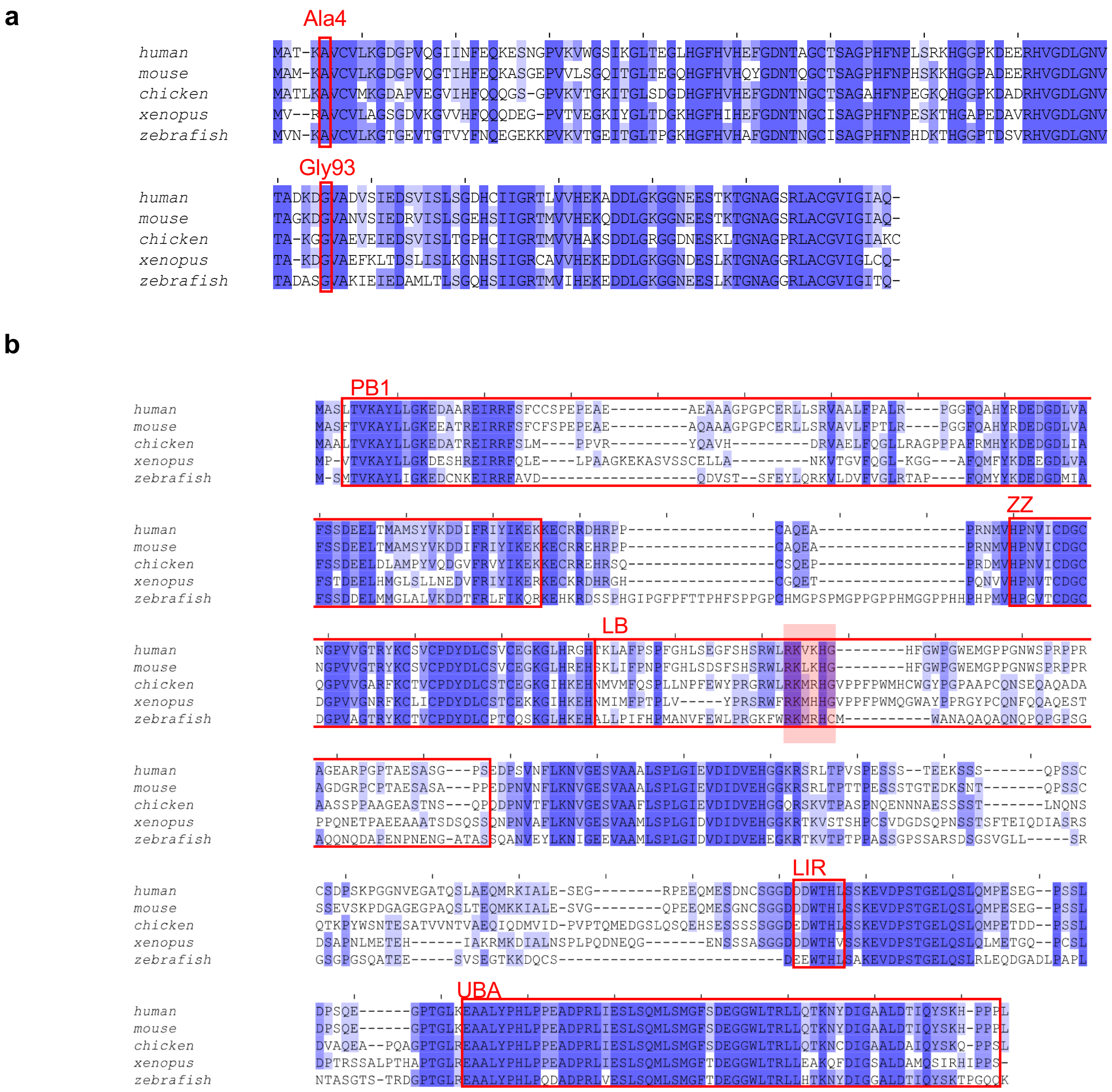

Supplementary Figure 6. Sequence alignment of SOD1 and p62 in vertebrates.

a SOD1 is also conserved in vertebrates. Human SOD1 shows high sequence identity (129/154, 84%) and similarity (137/154, 88%) with few gaps (0/154, 0%) to mouse; and similarly to chicken (115/155 (74%) identity; 133/155 (85%) similarity; 3/155 (1%) gap); xenopus (104/152 (68%) identity; 122/152 (80%) similarity; 2/152 (1%) gap); and zebrafish (108/154 (70%) identity; 118/154 (76%) similarity; 0/154 (0%) gap). Amino acids with known mutation in human patients (A4V and G93A) are conserved in all vertebrates investigated. **b** p62 is also conserved in vertebrates. Human p62 shows high sequence identity (401/442, 91%) and similarity (401/442, 94%) with few gaps (2/442, 0%) to mouse; and similarly to chicken (274/470 (58%) identity; 322/470 (68%) similarity; 47/470 (10%) gap); xenopus (246/461 (53%) identity; 312/461 (67%) similarity; 372/461 (8%) gap); and zebrafish (212/477 (44%) identity; 280/477 (58%) similarity; 72/477 (15%) gap). The regions corresponding to each p62 domain defined in study are indicated with red boxes. Background of each residue is colored as in supplementary Fig. 5a.

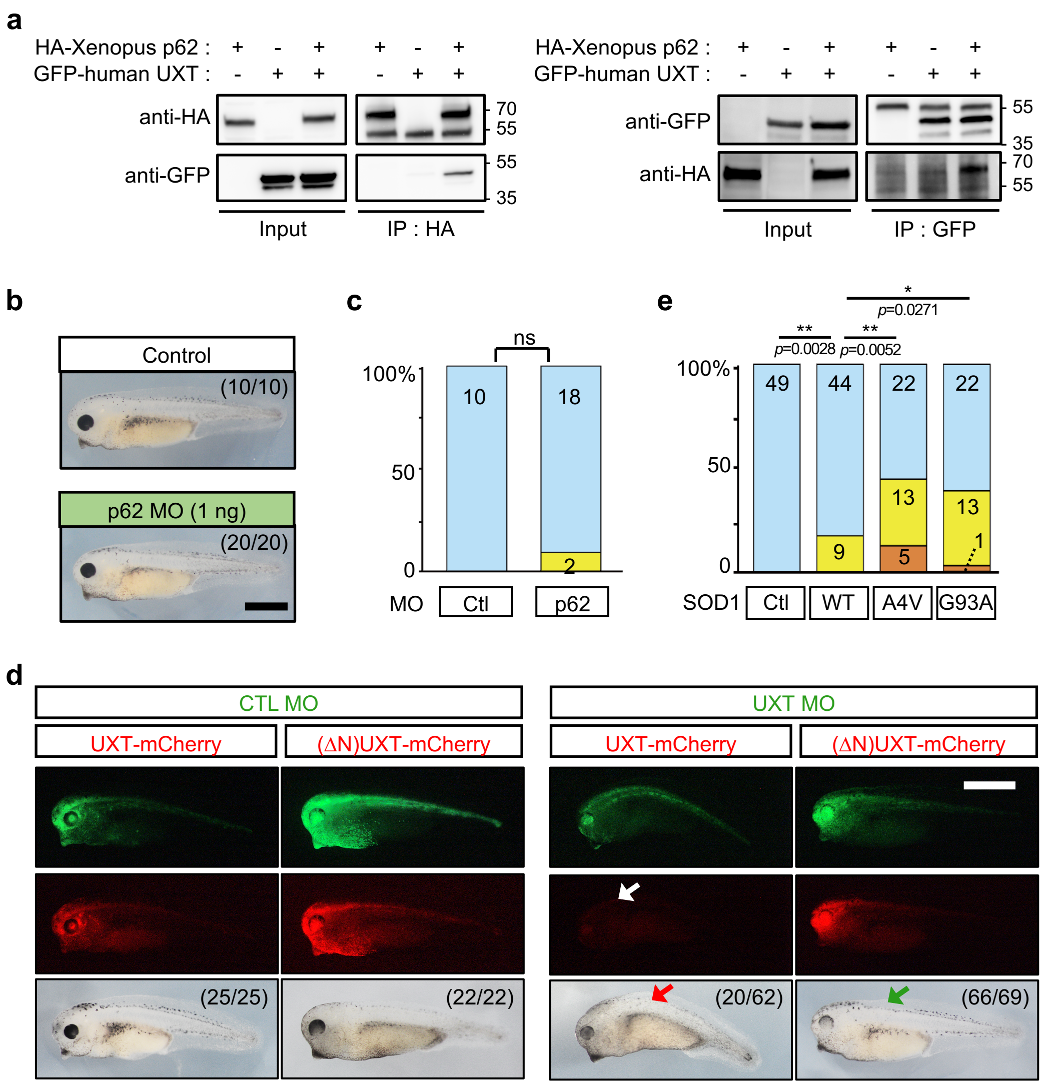

Supplementary Figure 7. Effects of knockdown of p62 and UXT in *Xenopus*.
a Interaction between human UXT and *Xenopus* p62 was confirmed by reciprocal co-immunoprecipitation experiments. **b** No apparent morphological change appears when p62 morpholino is injected to 4 blastomeres at 8 cells stage. The numbers indicate number of embryos with normal embryos / number of injected embryos analyzed. MO, morpholino. Scale bar, 1 mm. **c** Knock down of p62 do not induce significant difference in motor function at stage 43, when the most of behavioral assays in this study were performed. Cyan and yellow bars indicate normal and reduced swimming responses, respectively. The Y axis indicates the percentage of tadpoles, and the numbers indicate the number of animals. Ctl, control; MO, morpholino. ns, not significant (two-sided Fisher's exact test). **d** UXT MO-induced phenotype is specifically caused by the loss of UXT proteins and can be rescued by the MO-resistant UXT mutant. UXT-mCherry RNA or the (ΔN)UXT-mCherry RNA, which lacks the first 15 bases targeted by the MO, were injected with control morpholino (Ctl MO) or UXT MO. The amounts of injected MOs were identical, which can be seen in green fluorescence from the fluorescein tagged to MOs. UXT-mCherry expression was specifically blocked by UXT MO (white arrow). Most of these embryos displayed developmental defects (42 out of 62), suggesting that UXT MO inhibited the expression of the endogenous UXT in addition to the UXT-mCherry RNA (red arrow). This UXT MO-induced phenotype was completely rescued by the MO-resistant UXT mutant, (ΔN)UXT-mCherry RNA (green arrow). The numbers indicate the number of embryos with normal embryos / the number of injected embryos analyzed. Scale bar, 1 mm. **e** Overexpression of the wild type SOD1 (SOD1 WT) induces a small motor deficit. Cyan, yellow, and orange bars represent normal, reduced, and no swimming responses, respectively. The Y axis indicates the percentage of tadpoles, and numbers indicate the number of animals. *, $p < 0.05$; **, $p < 0.01$ (two-sided Fisher's exact test).

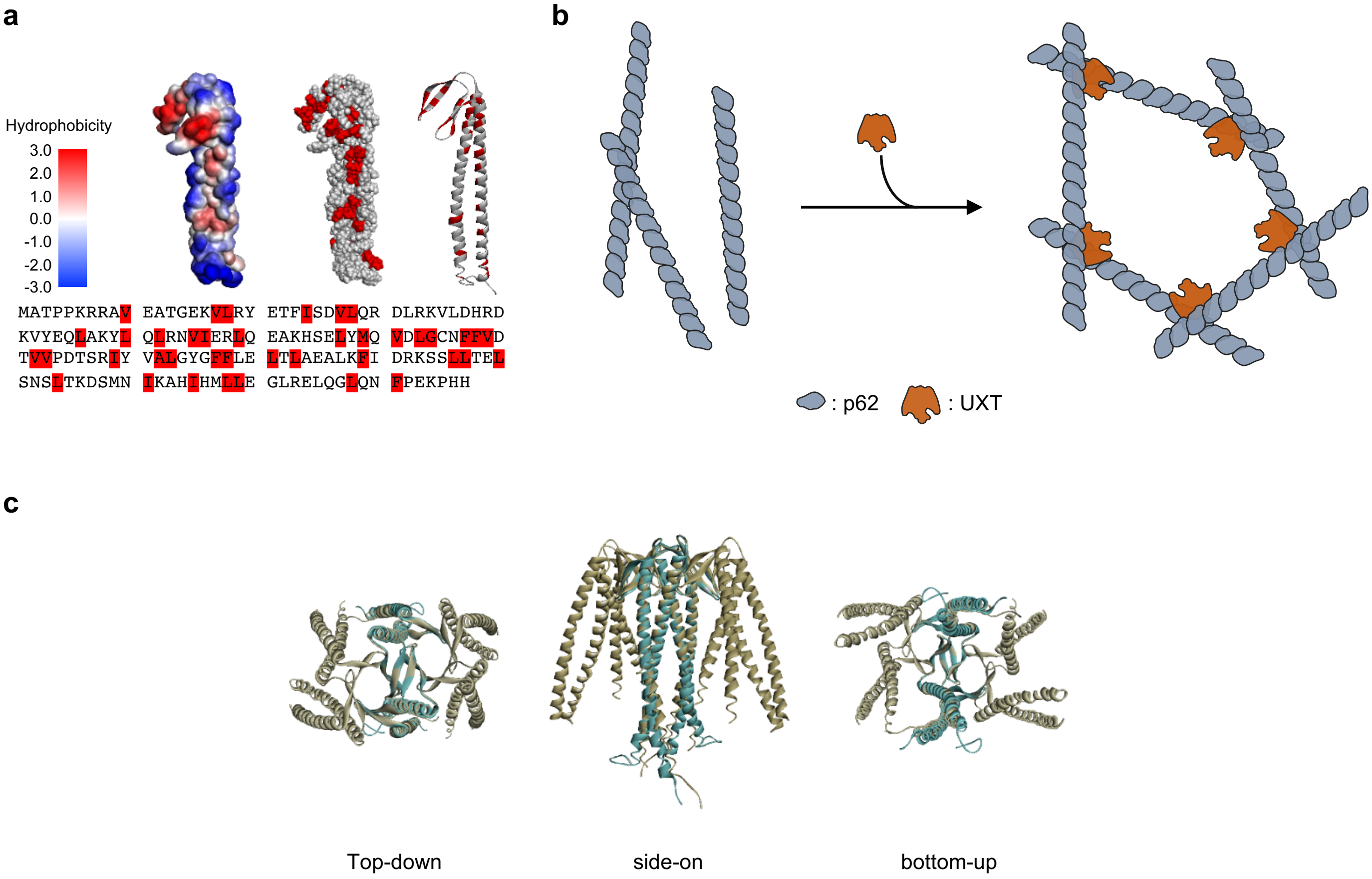

Supplementary Figure 8. UXT structure prediction and comparison with the prefoldin α subunit.
a Structural model of UXT is predicted from i-TASSER, which detects structure templates by a technique called threading. Based on more than 10 threading templates having fold-level homology with UXT, UXT structure model was predicted. Top-ranked threading templates were prefoldin subunits (PDB: 2zdiC, 2zdiA). The whole protein sequence and a predicted UXT structure (electrostatic potential mapped on the surface, atomic structure, ribbon diagram) are shown. Hydrophobic amino acid sequences are indicated in red in both the sequence and structure. **b** Schematic diagram of the possible role of UXT in generating a high-order p62 oligomer. **c** Structural match of the predicted UXT structure (cyan, predicted with i-tasser server) with the prefoldin hexamer (beige, 2zdi). For hexamer structure prediction, crystal structure of prefoldin from Pyrococcus horikoshii OT3 (PDB : 2ZDI) was modified with UCSF Chimera by substituting predicted UXT structural model for prefoldin subunit alpha (PDB : 2zidC). Top-down, side-on, and bottom-up views are shown.
