# Supplementary material for: UXT chaperone prevents proteotoxicity by acting as an autophagy adaptor for p62-dependent aggrephagy
Source: Nat Commun. 2021 Mar 29;12:1955. doi: 10.1038/s41467-021-22252-7 (PMC8007730; doi:10.1038/s41467-021-22252-7)
Supplement: Supplementary file 4 — Supplementary Code 1 [file 41467_2021_22252_MOESM4_ESM.zip › Supplementary code 1/README.pdf]

Our custom code 'ap.m' automatically selects and calculates aggregates/clusters index from **a cell in 16-bit TIF image**. 'ap.m' code was run in MATLAB R2019b (Mathworks, version 9.7.0.1261785). Note that all files – 'ap.m' and TIF images to be analyzed – should be in a same folder. Here we submitted a single compressed zip folder named 'code.zip' that contains all necessary contents for testing the code except MATLAB software.

'ap.m' code has been tested on Windows 10 version 1903 and macOS High Sierra version 10.13.6, and typical installation time on a normal desktop computer is about half an hour long.

1. MATLAB R2019b can be downloaded from http://www.mathworks.com/downloads/.
   Complete installation requires a license, but 30-day trial version is also available in the website. Please note that 'Image Processing Toolbox' is a necessary package for operating the 'ap.m' code. Select 'Image processing toolbox' for installation.
2. Test the code running with test samples – 'test sample1.tif' and 'test sample2.tif'. The average running time on a normal computer is about 3min. Running process and result are described in detail in Supplementary Figure 2.

   2-1. Run MATLAB software and move to the folder 'code'.

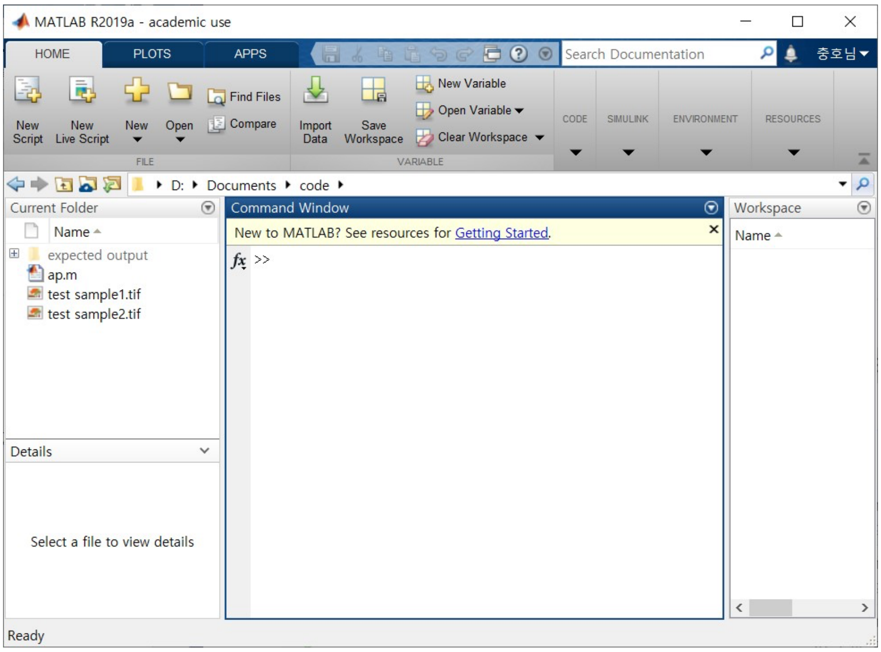

2-2. Run the ‘ap.m’ by enter ‘ap’ in command window.

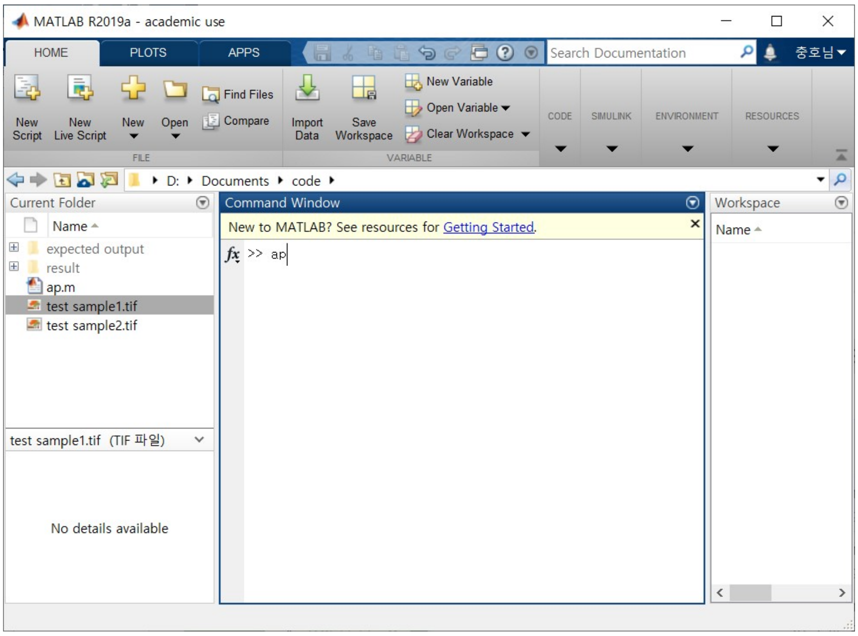

2-3. MATLAB will show an 8-bit image (Figure 1) from the first sample TIF file. Select background by click the left mouse button several times to make a closed region in the background, and complete selection by double click the selected region. (8-bit image is not used for calculating fluorescence intensity. Instead, it is used just for displaying the image.)

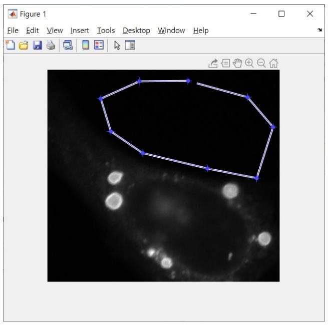

2-4. MATLAB will show 8-bit image again (Figure 2). Then, select the cell area and complete selection as in 2-2.

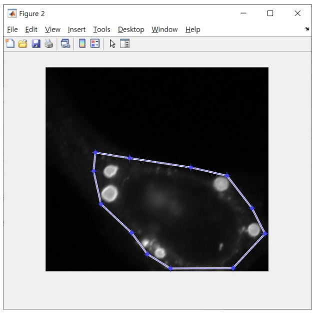

2-5. MATLAB will display a binary image (Figure 3) indicating the region of

clustering/aggregation with white.

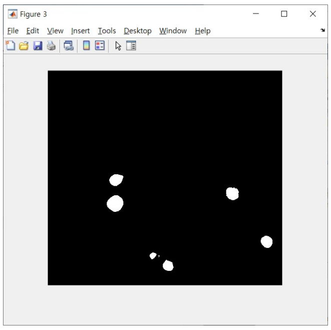

2-6. To continue the analysis with next image, enter ‘y’ (or just enter) and repeat the procedures on the next image. If there is a mistake during the analysis, enter ‘n’ and repeat the same procedures on the same image again. When analyses of all TIF image files in the folder are finished, the code will automatically stop.

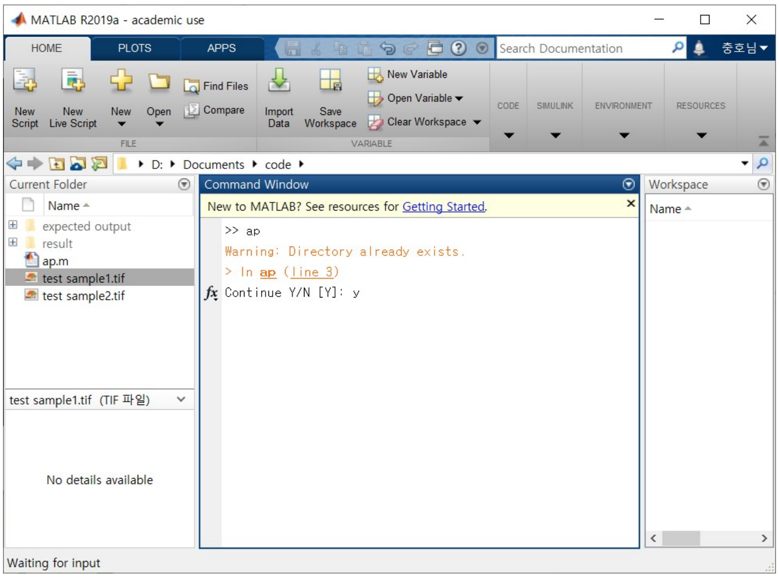

2-7. When finished, the binary images indicating the region of clustering/aggregation with white will be saved in ‘result’ folder. Sum of fluorescence intensities in the selected region (clustering/aggregation), the mean fluorescence intensities in the cell area, and the cluster index for each cell image will be saved in the automatically generated ‘result.xls’ file. Please see the legend for Supplementary Figure 2 for more information.

We tested the code and the expected results were put in the ‘expected output’ folder.
