# Supplementary figures and images for: UXT chaperone prevents proteotoxicity by acting as an autophagy adaptor for p62-dependent aggrephagy
Source: Nat Commun. 2021 Mar 29;12:1955. doi: 10.1038/s41467-021-22252-7 (PMC8007730; doi:10.1038/s41467-021-22252-7)

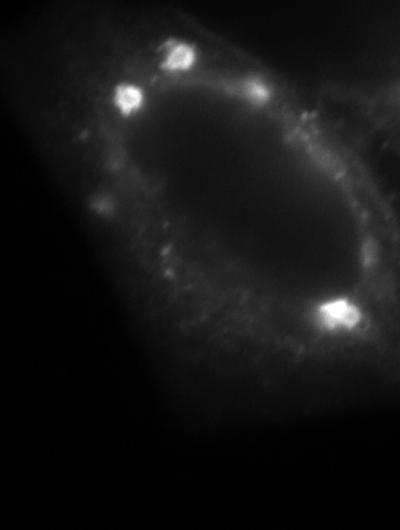

Supplement: Supplementary file 4 — Supplementary Code 1 [file 41467_2021_22252_MOESM4_ESM.zip › Supplementary code 1/test sample2.tif]

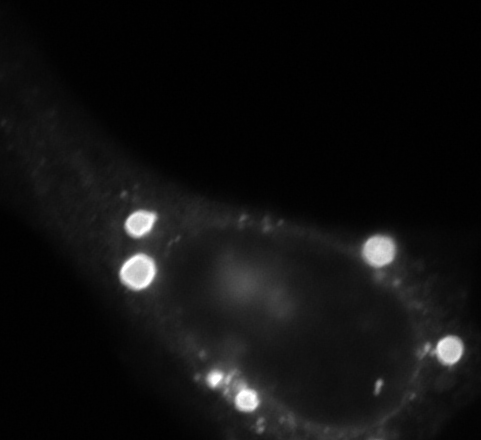

Supplement: Supplementary file 4 — Supplementary Code 1 [file 41467_2021_22252_MOESM4_ESM.zip › Supplementary code 1/test sample1.tif]

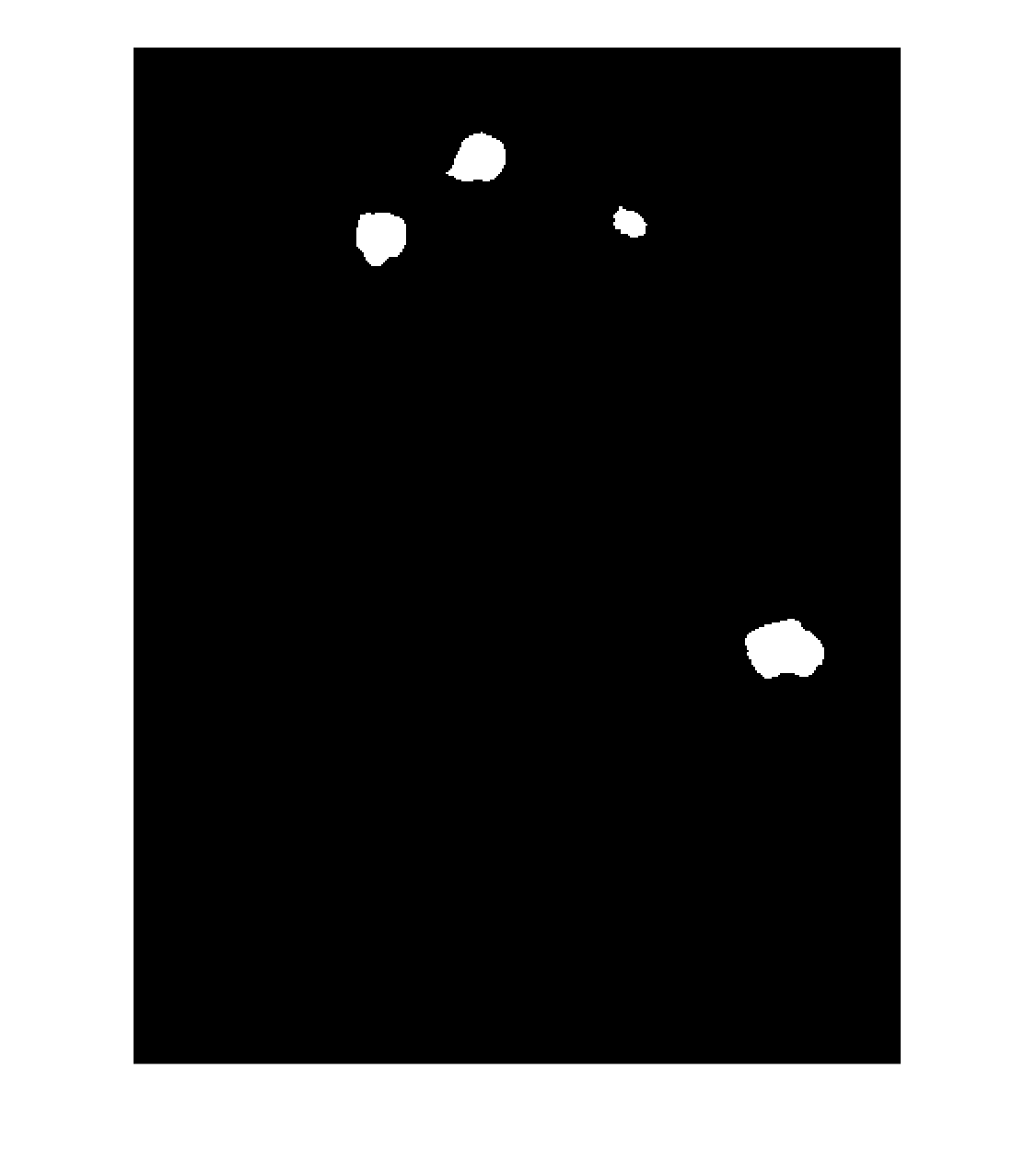

Supplement: Supplementary file 4 — Supplementary Code 1 [file 41467_2021_22252_MOESM4_ESM.zip › Supplementary code 1/expected output/result/test sample2.tif]

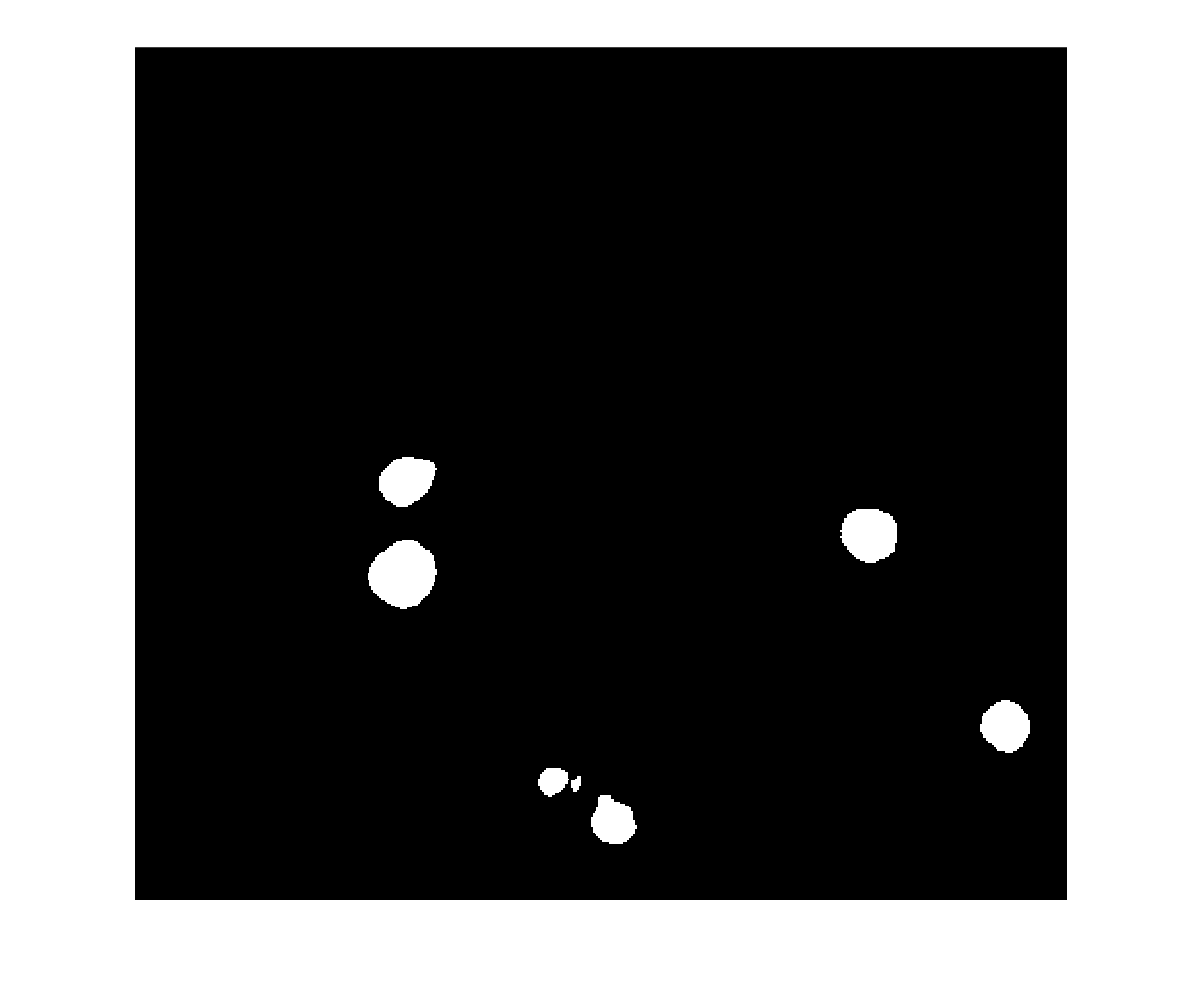

Supplement: Supplementary file 4 — Supplementary Code 1 [file 41467_2021_22252_MOESM4_ESM.zip › Supplementary code 1/expected output/result/test sample1.tif]
