# Supplementary material for: UXT chaperone prevents proteotoxicity by acting as an autophagy adaptor for p62-dependent aggrephagy
Source: Nat Commun. 2021 Mar 29;12:1955. doi: 10.1038/s41467-021-22252-7 (PMC8007730; doi:10.1038/s41467-021-22252-7)
Supplement: Supplementary file 12 — Description of Additional Supplementary Files [file 41467_2021_22252_MOESM12_ESM.docx]

Description of Additional Supplementary Information

Title: Supplementary Movie 1.

Description: Time-lapse image of MG132-induced apoptotic blebbing in DRG neurons. (a, b) DRG neurons subjected to lentiviral transduction of mCherry control (a) or mCherry-fused UXT (b) were treated with MG132 and observed at 20 min intervals for 28 h. DRG neurons transduced with each lentiviral construct are indicated with red asterisks. Membrane blebs are indicated with arrowheads (red, blebs of transduced cells; white, blebs of nontransduced cells). (c) DRG neurons treated with MG132 were first observed in the real time setting, and the cells were fixed and stained with anti-cleaved caspase-3 followed by anti-rabbit IgG-Alexa-488. The time lapse phase contrast images were aligned with the cleaved caspase-stained fluorescence image.

Title: Supplementary Movie 2.

Description: Time-lapse image of SOD1(A4V) aggregates formation. Time-lapse images from the experiment described in Fig. 4d are shown.

Title: Supplementary Movie 3.

Description: Scoring motor function in Xenopus. Behavior was classified into the following three categories: normal, reduced, and no swimming response.

Title: Supplementary Movie 4.

Description: Age-dependent loss of motor function induced by SOD1(A4V) expression. Responses of the same embryo recorded at stage 28 (day 2) and stage 43 (day 4). Two examples are shown in which embryos display a complete escape response at stage 28 (normal response) and lose this ability at stage 43 (no response).

Title: Supplementary Movie 5.

Description: Complete loss of swimming ability in SOD1(A4V)-expressing tadpoles. In this example, the tadpole has lost its ability to swim but is alive, which is evident from its normal heartbeat.
